# Application of Mixing Rules for Adjusting the Flowability of Virgin and Post-Consumer Polypropylene as an Approach for Design from Recycling

**DOI:** 10.3390/polym14132699

**Published:** 2022-06-30

**Authors:** Ines Traxler, Christian Marschik, Manuel Farthofer, Stephan Laske, Joerg Fischer

**Affiliations:** 1 Competence Center CHASE GmbH, Altenberger Strasse 69, 4040 Linz, Austria; christian.marschik@chasecenter.at; 2 Institute of Polymeric Materials and Testing, Johannes Kepler University Linz, Altenberger Strasse 69, 4040 Linz, Austria; manuel.farthofer@jku.at (M.F.); joerg.fischer@jku.at (J.F.); 3 Greiner Packaging International GmbH, Gewerbestrasse 15, 4642 Sattledt, Austria; s.laske@greiner-gpi.com

**Keywords:** polypropylene recyclate, mixture rules, polymer blends, MFR adaption, predictive model, design from recycling

## Abstract

To enable the use of recyclates in thermoformed polypropylene products with acceptable optical appearance and good mechanical stability, a multilayer structure of virgin and recycled material can be used. When producing multilayer films with more than two layers, the used materials should have similar melt flow properties to prevent processing instabilities. In the case of a three-layer film, post-consumer recyclates are often hidden in the core layer. Due to the inconsistent melt flow properties of post-consumer recyclates, the adjustment of the melt flow properties of the core layer to those of the outer layers has to be realized by blending with virgin materials. In order to understand the effect of mixing with a virgin material with a certain pre-defined melt flow rate (MFR), material mixtures with different mixing partners from various sources were realized in this study. Hence, the pre-defined virgin material was mixed with (i) virgin materials, (ii) artificial recyclates out of a mixture of different virgin materials, and (iii) commercially available recyclates. These blends with mixing partner contents ranging from 0–100% in 10% increments were prepared by compounding and the MFR of each mixture was determined. For a mathematical description of the mixing behavior and furthermore for a proper MFR prediction of the material mix, existing mixing rules were tested on the three pre-defined sample groups. Therefore, this paper shows the applicability of different mixing rules for the prediction of the MFR of material blends. Furthermore, a new mixing rule was developed using symbolic regression based on genetic programming, which proved to be the most accurate predictive model.

## 1. Introduction

Plastics recycling gained a lot of public, governmental, and scientific interest within the last few years [1,2,3,4]. According to the EU directive 2018/852, at least 50% and 55% of plastic packaging waste must be recycled annually until the end of 2025 and 2030, respectively [5]. In addition to the design for recycling approach, which specifies that products must be designed in a way that they can be recycled (e.g., mono-material), there is also the design from recycling approach [6]. A promising way of design from recycling without sacrificing visual quality and mechanical stability while using the required amount of plastic recyclates is to produce products with a multilayer structure to encapsulate the recyclates. Recyclates, which often have a grayish or greenish appearance and are therefore not as attractive as transparent, clear white, or brightly colored materials, can be wrapped in pigmented top layers [7]. Furthermore, migration can be weakened by the use of multilayer structures, which could help recyclates to be applicable for food contact products [8].

A huge drawback of post-consumer polyolefin recyclates is the strongly fluctuating melt flow properties, which depend on the constantly changing waste input streams caused by different compositions of the waste fractions. Moreover, this is associated with fluctuating property profiles of the recyclates. Varying melt flow rates (MFRs) are often stated as a problem in the recycling of polypropylene (PP), which is widely used in the plastics packaging industry with a share of around 20% [9]. Due to the MFR variations, recyclate suppliers often only provide a wide range for the MFR in their data sheets [10]. While, for thermoforming of packaging products, PP needs a rather low MFR of around 4 g/10 min (230 °C/2.16 kg), when processing PP by blow and cast film extrusion, grades with MFR of up to 11 g/10 min are used. Depending on the wall thickness of the product and with focus on energy consumption savings, for injection molded products PP with MFR of 3 to 100 g/10 min is utilized [11]. Packaging products such as films, trays, cups, bottles, buckets, and containers are produced with a wide range of melt flow properties. If collected in a separate collection, at their end-of-life they are mixed together in the lightweight packaging waste fraction. Even when separated into their plastics recycling codes in the further steps of waste management, a mixture of materials with different MFRs is inevitable. The material mix leads to averaged and rather high MFR values of commercial PP recyclates. Additionally, material degradation within the previous life cycle could also negatively influence the property profile (e.g., increase in MFR) of the PP recyclate. The high MFR values resulting due to material mixtures and material degradation could result in poor performance in extrusion and thermoforming as low MFR values are required.

The MFR in general is an important parameter in polymer processing and due to its simplicity widely used as an input quality assurance parameter in industry. In contrast to the viscosity, which can be evaluated by a number of rheological measurements (e.g., parallel plate rheometer or high-pressure capillary rheometer) and is expressed as multipoint data, the MFR can be expressed as a single point value with a minor dependency on temperature and shear rate given solely by the used testing temperature and weight. The standards ISO 1133 and ASTM D1238 are generally used to evaluate the MFR. It is determined by extruding the molten polymer at a fixed temperature and weight, which represents a certain shear rate, through a standardized die [12,13,14]. The MFR of polymers is basically dependent on their molar mass and its distribution. Additionally, additives can influence the MFR. The higher the molar mass the lower the MFR. Furthermore, branching and the presence of plasticizers affect its magnitude [15]. Especially in PP, the MFR is strongly affected by material degradation. PP is influenced by thermo-oxidative and UV light-initiated degradation in warm and bright environments leading to chain scission and thus to molar mass reduction and MFR increase [16]. This effect in combination with the influence of material changes on thermal and mechanical properties is already discussed in the literature by parametrization of different formulations [17,18,19,20,21,22,23]. Nevertheless, this paper discusses MFR value changes as they are of most importance for machine operators. To overcome the inferior mechanical properties, virgin materials are added in terms of blends but also by using multilayer structures or by changing the product design.

The coextrusion process of multilayer films is rather sensitive to MFR fluctuations. Multilayer films are used, for example, to minimize migration and permeation [24]. Three- and five-layer films are mostly used for this purpose. To prevent flow instabilities, the middle layer of a multilayer structure is typically supposed to have either the same or a lower MFR compared to its embedding layers. Otherwise, viscous encapsulation effects may occur, where the lower viscous material encapsulates the material with the higher viscosity during the extrusion process [25]. This effect, which has been widely studied in the literature [26,27,28,29], typically occurs in stratified flows in rectangular cross sections.

In particular, post-consumer recyclates often exhibit strongly fluctuating MFR values from one lot to another. As a result, recyclate manufacturers usually indicate only rough MFR ranges in their data sheets. Therefore, attention must be paid to the MFR value of the specific lot of the recyclate. In an ideal world, it is stated in the lot certificate of the recyclate, which is required due to recycling material specifications.

Mixing rules can be used to calculate the MFR of binary blends. For a large number of mixing rules, only the MFR values and the weight fractions of the mixing components must be known to evaluate the MFR of the mixture. While these mixing rules have been intensively analyzed for a variety of virgin polymers [30,31], recyclates have received less attention. In this work, the accuracy of selected mixing rules in predicting the MFR of binary PP blends was investigated. To this end, samples were produced by compounding at least two different PP types. The melt flow behavior of one component was changed stepwise from 8 to 25 g/10 min, whereas the other component was fixed at an MFR of 4 g/10 min. The contents of the components were varied between 0% and 100% with an increment of 10%. PP homopolymers, PP copolymers, and post-consumer PP recyclates were used to cover a large variety of possible combinations. The overall objective was to assess the accuracy of selected existing mixing rules and to find a relationship, which can be used universally for various PP blends with a particular focus on recyclates. Based on the observed relationships and the measured experimental data, a new mixing rule is presented, using symbolic regression based on genetic programming.

## 2. Experimental

### 2.1. Materials and Characterization

Five polypropylene (PP) homopolymer grades, two PP copolymer grades, and two post-consumer polypropylene recyclate (rPP) grades were used for this study. The materials were supplied by Borealis (Vienna, Austria), Lyondell Basell (Rotterdam, The Netherlands), and mtm plastics (Niedergebra, Germany). The MFR values from the data sheets were measured according to ISO 1133 with a temperature of 230 °C and a weight of 2.16 kg. In the following, materials are abbreviated according to (i) their origin (i.e., v for virgin and r for recyclate), (ii) their type of virgin polypropylene (i.e., H for homopolymer or B for block copolymer, and M for mix), and (iii) their MFR value in the data sheet. Table 1 shows an overview of the materials analyzed in this work.

Eight material mixtures were defined, as explained in Table 2, where $MFR_{1}$ and $MFR_{2}$ indicate the MFR values of the first and the second component, respectively. To decrease the MFR of the blend, the material vH4 with an MFR of 4 g/10 min was used as a blending partner for all material mixtures. As a first material mixture group, all homopolymer grades (vH8, vH12, vH20, and vH25) were mixed with the blending partner vH4. In the second group, artificial recyclates consisting of PP homopolymers and the PP block copolymer vB8 were mixed with vH4. These artificial recyclates were mixed together to simulate a PP waste mix consisting of various different PP grades. However, by formulating an artificial recyclate, the composition contents are well known which enable more accurate conclusions. In the third material mixture group, mixtures were produced using the post-consumer recyclates (i.e., r16 and r27) and vH4. In the fourth group, two model validation mixtures were defined. For the applicability in industry, weight percentages were applied for the mixtures instead of moles as proposed for most of the mixing rules.

All material mixtures were produced using a Leistritz ZSE 18 MAXX compounder (Leistritz Extrusionstechnik GmbH, Nuremberg, Germany). During sample production, the weight percentages of the first and second blend components were increased and decreased by 10%, respectively (see Table 3). In addition, 0 and 100% samples were compounded to consider material degradation of the polymer during the compounding process. In total, 11 mixtures with component contents ranging from 0% to 100% were produced for each material combination of the four material mixture groups. For compounding, a temperature of 210 °C, a screw speed of 400 rpm, and a throughput of 8 to 10 kg/h were used. To ensure proper mixing, screws with nine kneading and three mixing elements were used. The extruded strands were cooled in a cold-water bath and granulated with a strand pelletizer.

The MFR values of all material mixtures were determined using a Zwick Roell Aflow plastometer (ZwickRoell GmbH & Co. KG, Ulm, Germany) with around 4 g of granules. The measurements were performed according to ISO 1133 [12]. According to the standard test conditions for polypropylene, a temperature of 230 °C and a weight of 2.16 kg were utilized for the measurements. The pre-heating time was set to 300 s. Six extrudates were produced per measurement with cuts after 5 mm of piston movement. The weight of the six individual extrudates was determined using a Sartorius Quintix laboratory scale (Sartorius AG, Goettingen, Germany) with an accuracy of four digits after the decimal point in grams. Multiple measurements were performed for each sample.

### 2.2. Mixing Rules

Table 4 shows the selected mixing rules investigated in this research work. The less viscous component (i.e., higher MFR) is labeled as $MFR_{1}$, while the more viscous component (i.e., lower MFR) is labeled as $MFR_{2}$. The weight fractions of the respective components in the mixture are named $x_{1}$ and $x_{2}$, where the sum must be equal to 1. The subscript indicates the affiliation to the less and more viscous components. The linear mixing rule is analyzed as a reference. The parameter n in the equation from Kendall and Monroe (K & M) is usually set to 3 or 3.4, referring to the articles by Gao and Li, Haley and Lodge, and Friedman and Porter [30,32,33]. In this paper, the constant was set to 3. In the mixing rule by Cragoe, the constant L is dependent on the viscosity of the observed liquid and it was set to 2000 as proposed by Gao and Li [32]. The constant C in the mixing rule of Walther was set to 0 [32].

The accuracy of the mixing rules in Table 4 in predicting our experimentally determined MFR values was evaluated using the mean absolute error (MAE, given in g/10 min) and the mean relative error (MRE, given in %), as shown in Equations (1) and (2), respectively. The calculated MFR ($MFR_{calc}$) was compared to the mean value of the experimental MFR ($MFR_{exp}$). With the help of the mean absolute error, it is not possible to assess whether the values are overestimated or underestimated by a mixing rule, which is why additional parity plots were used in the following sections. The coefficient of determination $R^{2}$ was used additionally to assess the accuracy of the mixing rules (Equation (3)). $MFR_{mean}$ is the mean of the experimental data.

(1) $$MAE=\frac{1}{N}\;\overset{N}{\underset{i=1}{\sum }}\left|MFR_{calc,i}-MFR_{exp,i}\right|$$

(2) $$MRE=\frac{1}{N}\;\overset{N}{\underset{i=1}{\sum }}\frac{\left|MFR_{calc,i}-MFR_{exp,i}\right|}{MFR_{exp,i}}$$

(3) $$R^{2}=1-\frac{\sum_{i=1}^{N}{\left(MFR_{exp,i}-MFR_{calc,i}\right)}^{2}}{\sum_{i=1}^{N}{\left(MFR_{exp,i}-MFR_{mean}\right)}^{2}}$$

## 3. Results

### 3.1. Modeling with Virgin Blends

**Figure 1:**
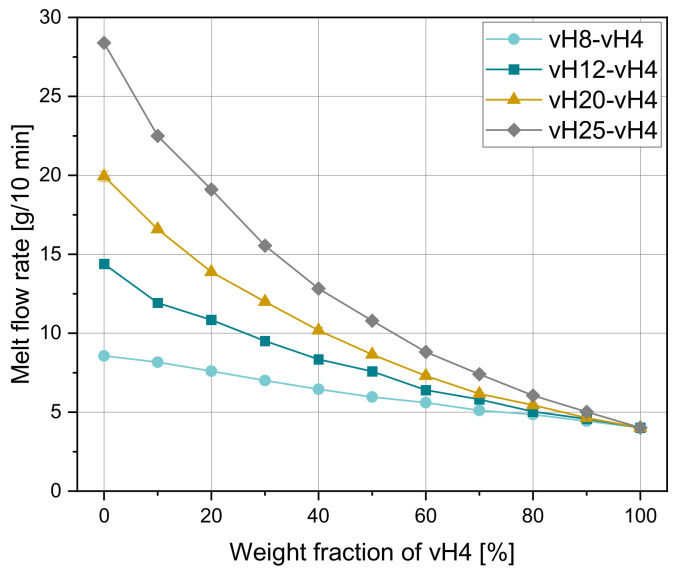
Melt flow rate depending on the weight fraction of vH4 of four virgin homopolymer mixtures vH8-vH4, vH12-vH4, vH20-vH4, and vH25-vH4.

This section deals with the mixing behavior of virgin PP homopolymers with different initial MFR values. As MFR values are single point data, one material mixture is presented as a single dot in Figure 1. However, for a better visualization, the MFR values of a certain material blend (e.g., vH25-vH4) are connected into a curve. Hence, trends of the mixing behavior are observable. The curves in Figure 1 show a linear decrease for vH8-vH4 and an exponential decrease for vH12-vH4, vH20-vH4, and vH25-vH4. In some cases, the MFR of the unblended material is slightly higher than stated in the data sheet as the material is damaged and degraded up to a certain amount during compounding. However, no trend on the influence of the initial MFR on the material degradation behavior due to compounding was deducible. The initial MFR values after compounding for vH12 and vH25 (leftmost points in the diagram) are 14.4 g/10 min and 28.4 g/10 min, compared to 12 g/10 min and 25 g/10 min as stated in the data sheets, respectively. The MFR values of the remaining materials vH4, vH8, and vH20 did not change significantly due to compounding. Error bars are drawn at the individual MFR values and are hardly visible due to the insignificant variation.

For all blends, the maximum MFR differences between the weight fractions of 0 and 100% of vH4 were calculated to be 4.6, 10.4, 15.9, and 24.4 g/10 min, respectively. For a large MFR difference of two unmixed homopolymers, the decrease in MFR of the mixture is more pronounced than for small differences. The smaller the difference, the more linear the relationship. In general, the slope is steeper in the left than in the right part of the curve, with the curve flattening out in the right part. The higher slope in the left part of the diagram is most likely due to the influence of molar mass and thus the number of entanglements. With lower molar mass, the flowability is increased due to easier disentanglement [42].

**Figure 2:**
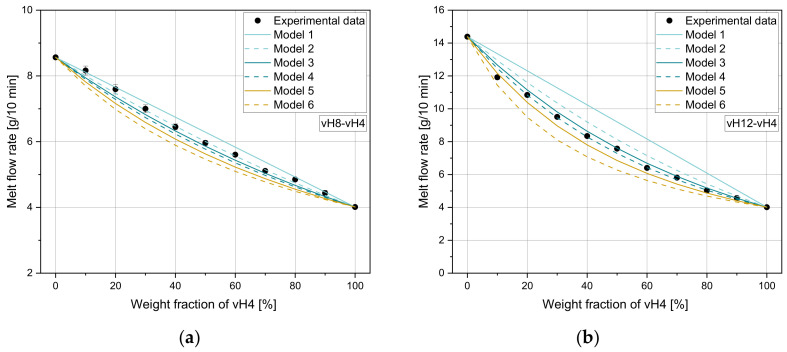

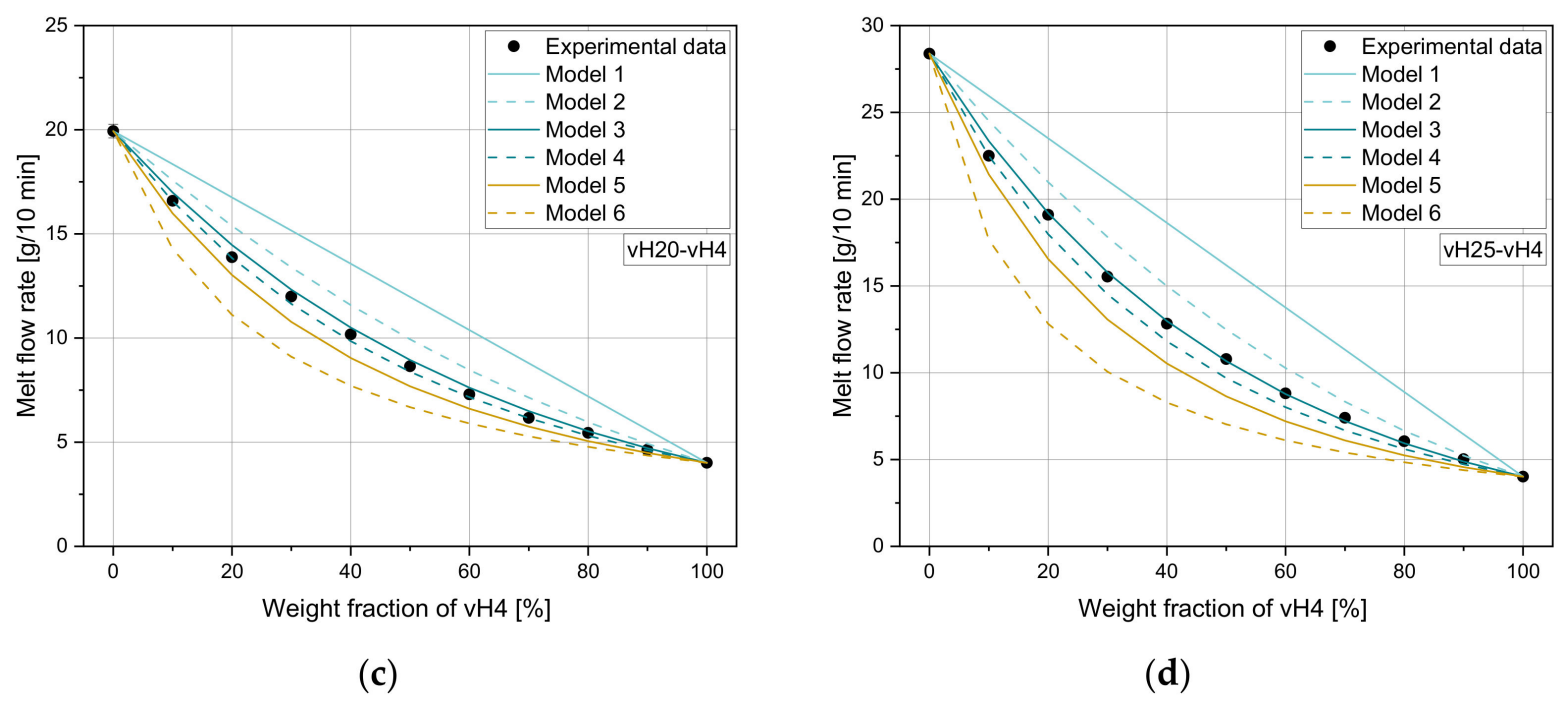
Application of six mixing rules on (**a**) vH8-vH4, (**b**) vH12-vH4, (**c**) vH20-vH4, and (**d**) vH25-vH4.

Figure 2a–d compare the experimental MFR values for all homopolymer blends with the calculated values according to the mixing rules in Table 4. For all blends, the measured values lie mostly between the predicted results according to model 3 and model 4, where model 3 overestimates and model 4 underestimates the measured MFR values. However, these two mixing rules show the best fit independent of the weight fraction of vH4. For small MFR differences (e.g., vH8-vH4), all mixing rules seem to be applicable (see Figure 2a), while for large MFR differences model 1 and model 6 lead to larger errors. These two mixing rules are quite accurate for very small weight fractions of vH4, while in the middle of the curves with weight fractions of around 50% of vH4, the deviations are more pronounced. Models 2 and 5 show a slightly worse performance for all investigated mixtures compared to model 3 and 4. In general, models 1 to 3 overestimate the experimental MFRs, while models 4 to 6 underestimate the experimental values.

**Figure 3:**
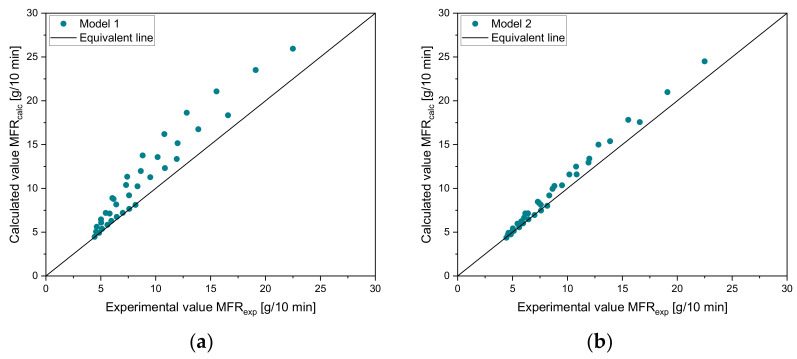

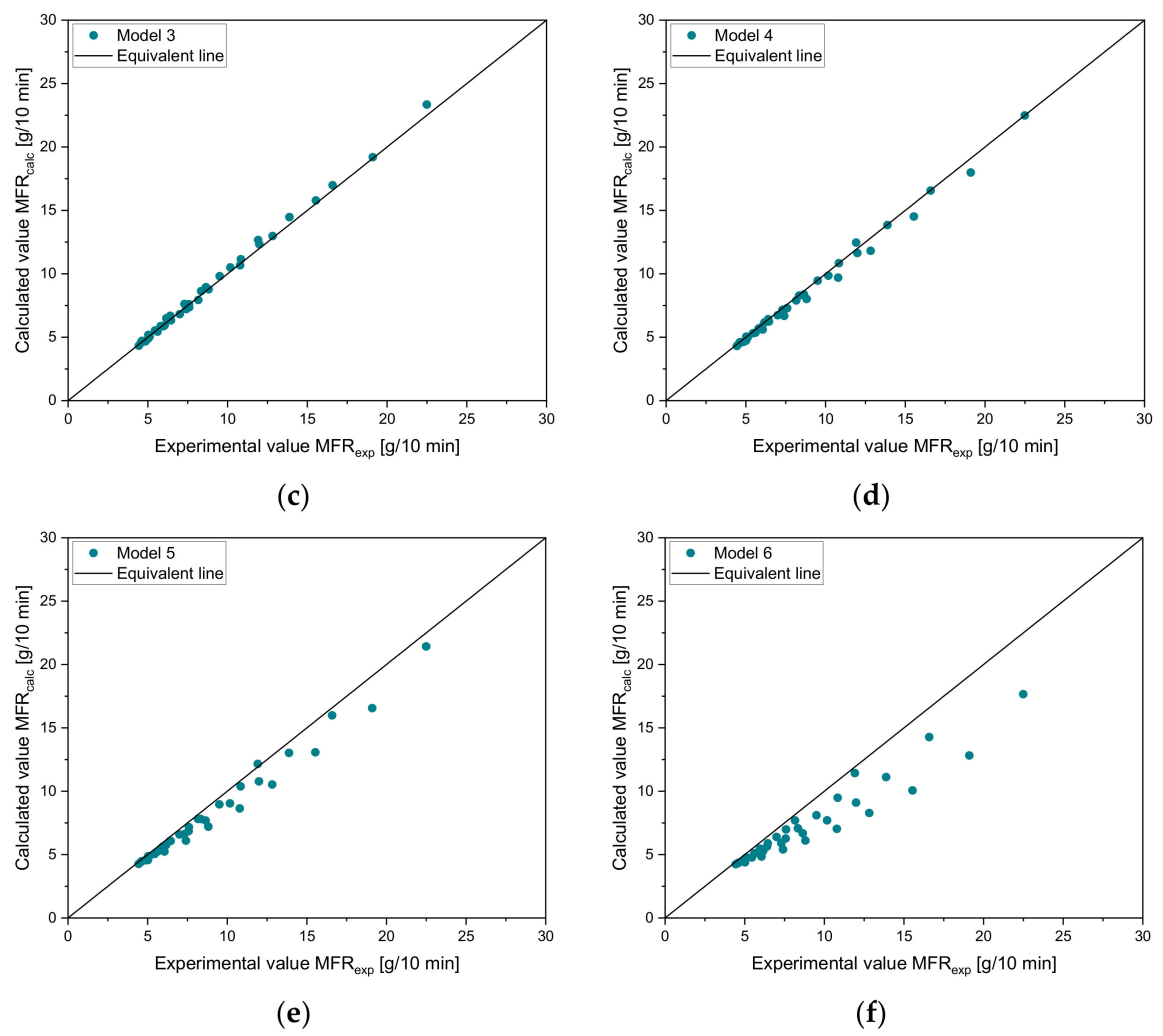
Parity plots for comparison of experimental and calculated values of virgin mixtures for six different mixing rules: (**a**) model 1, (**b**) model 2, (**c**) model 3, (**d**) model 4, (**e**) model 5, and (**f**) model 6.

By the use of parity diagrams, which are shown in Figure 3 for the four tested mixtures (vH8-vH4, vH12-vH4, vH20-vH4, and vH25-vH4), positive and negative changes in the mixing rules can be evaluated. If the results of both methods match exactly, then the point lies exactly on the 45° equivalent line. Larger distances to this line would indicate a worse accuracy of the mixing rule. Clearly, model 3 (see Figure 3c) and model 4 (see Figure 3d) show the best estimation of the calculated data, which is clearly indicated by the proximity of the data points to the 45° equivalent line. Again, the same trend in overestimation (i.e., positive change) and underestimation (i.e., negative change) of the other models is detectable with models 1 and 6 as the worst predictive models. Table 5 and Table 6 show (i) the MAE, (ii) the MRE, and (iii) the Pearson $R^{2}$ for all blends. The lowest error is shown in bold for every mixture. The higher the MFR difference, the worse the accuracy of the other models. For blend vH8-vH4, all mixing rules produce small errors with a minimum of 0.06 g/10 min for model 2. All remaining mixing rules also achieve satisfying results. In this case, the highest error was obtained from model 6 with an MAE of 0.38 g/10 min. For vH12-vH4 and vH20-vH4, model 4 delivered the best results. For vH2-vH4, by far the lowest errors were calculated using model 3.

### 3.2. Modeling with Artificial Blends

**Figure 4:**
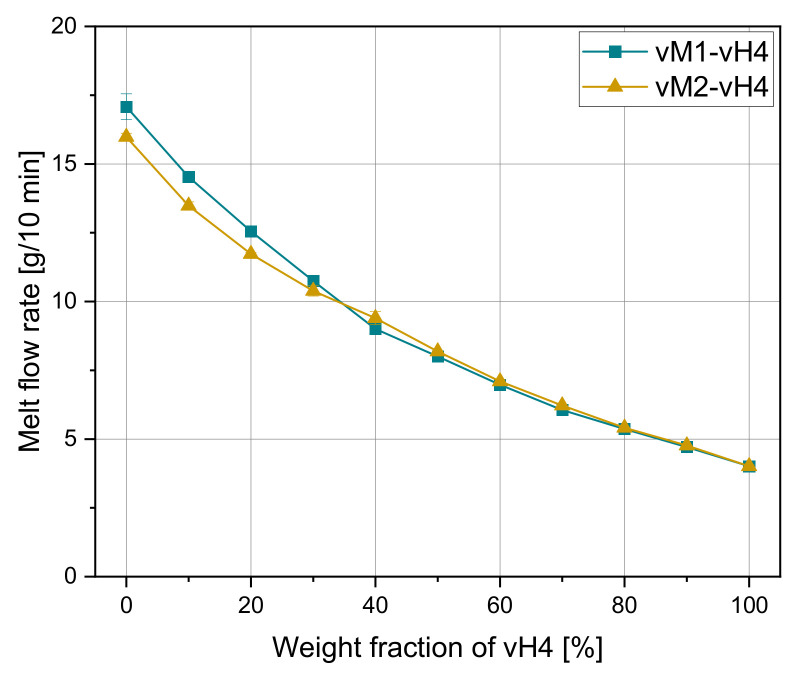
Melt flow rate depending on the weight fraction of vH4 of artificial mixtures vM1-vH4 and vM2-vH4.

Due to their good agreement with the experimental MFR values, only the results of the mixing rules model 3 and model 4 are shown for the artificial blends. In Figure 4, the curves for both artificial mixtures vM1-vH4 and vM2-vH4 are shown. Mixture vM2-vH4 shows a slightly lower MFR at 0% of vH4 due to the quite low MFR of the block copolymer vB8 (8 g/10 min). Interestingly, at around 35% the two curves intersect. This might come from the influence of ethylene phases in the heterophasic block copolymer, which results in a lower slope of vM2-vH4.

**Figure 5:**
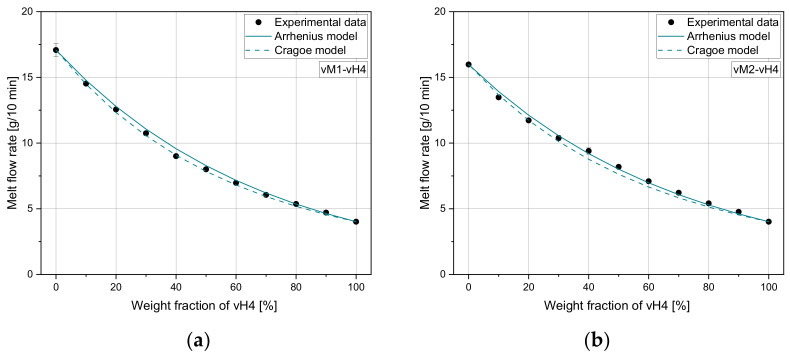
Application of Arrhenius and Cragoe mixing rules to (**a**) vM1-vH4 and (**b**) vM2-vH4.

Figure 5 demonstrates the predicted MFR values according to the mixing rules of models 3 and 4 for vM1-vH4 and vM2-vH4. The measured data from vM1-vH4 are in the middle of both calculated curves. For vM2-vH4, both mixing rules show a good agreement in the region from 0–30%, while the values in the region from 40–100% are slightly underestimated.

**Figure 6:**
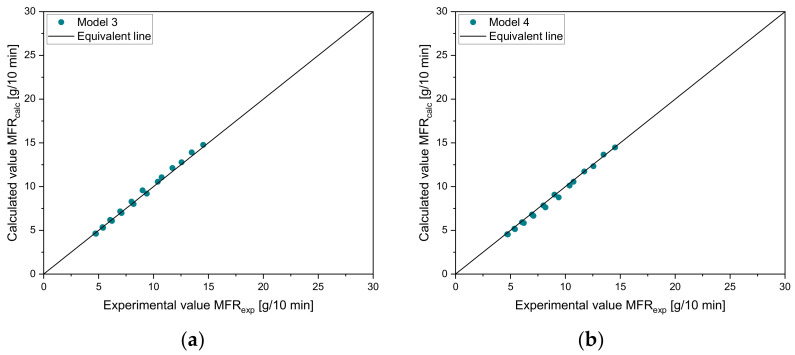
Parity plots for comparison of experimental and calculated values of artificial recyclates for (**a**) model 3 and (**b**) model 4.

The parity plots for model 3 and model 4 using the data from sample set 2 are shown in Figure 6. Both models show a good agreement with the data points. In Table 7, the error measures are depicted. For vM1-vH4, model 4 delivers more accurate results, while for vM2-vH4, model 3 delivers a lower error. Therefore, the selection of these two rules of mixtures is confirmed. These results are in good agreement with Gao and Li [32].

### 3.3. Modeling with Recyclate Blends

**Figure 7:**
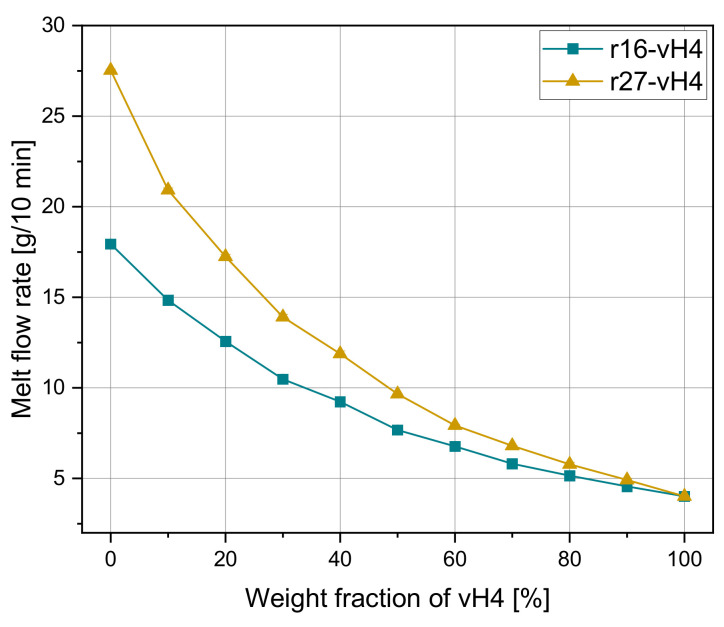
Melt flow rate depending on the weight fraction of vH4 of recyclate blends r16-vH4 and r27-vH4.

In the next step, models 3 and 4 were applied to recyclate–virgin blends. The experimental MFRs of the recyclate blends r16-vH4 and r27-vH4 are shown in Figure 7 as a function of the weight fraction of vH4. The blend r16-vH4 starts at 18 g/10 min and the blend r27-vH4 starts at 27.5 g/10 min. Similar to the previous mixtures, the exponential MFR decrease is obvious for rising weight fractions of the blending partner vH4. Again, almost no standard deviations of the individual measurement points are visible. Langwieser et al. [20] discussed the influence of multiple processing (e.g., plastics recycling) on various properties such as mechanical and thermal properties. In particular, the strain at break values decrease significantly with increased recycling loops. The increase in the MFR value is directly related to the decrease in the mean molar mass. Besides polymer degradation, the influence of contamination from other polymers as well as of inorganic particles must be considered. Therefore, in contrast to virgin PP, the processing of recyclates is usually more complicated.

**Figure 8:**
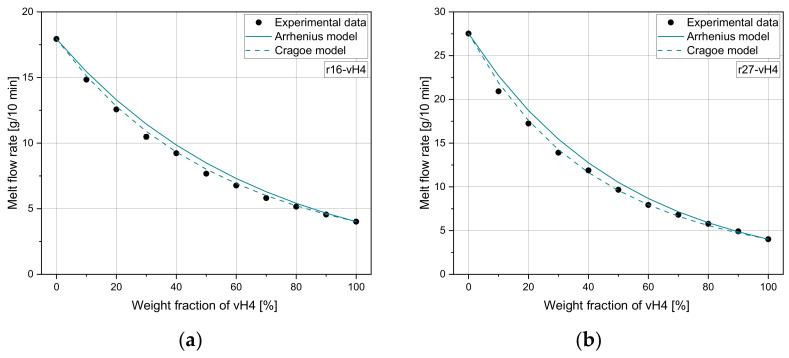
Application of model 3 and model 4 to (**a**) r16-vH4 and (**b**) r27-vH4.

**Figure 9:**
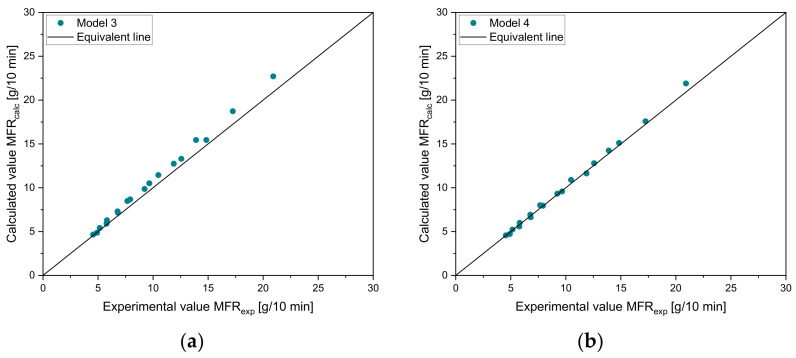
Parity plots for comparison of experimental and calculated values of commercial recyclates for (**a**) model 3 and (**b**) model 4.

Figure 8 and Figure 9 show the performance of model 3 and model 4 for both recyclate blends. For both blends, it can be seen that both mixing rules overestimate the measured values, especially for low vH4 weight fractions. 

As can be seen in Table 8, the MAE of 0.16 and 0.23 g/10 min for r16-vH4 and r27-vH4, respectively, for the mixing rule according to model 4 is very low. There is still minor potential for improvement in the application-relevant range of 0–50%. Nevertheless, the accuracy in predicting the MFR of recyclate blends is in the same order of magnitude as determined for the pure homopolymer mixtures and the artificial blends. Additionally, the Pearson $R^{2}$ shows the same trend as the mean errors ranging from 0.994 to 0.999. Therefore, the applicability of well-established mixing rules for recyclates is corroborated. In contrast to other literature, model 4 seems to be of higher importance for recyclates than the most widely used model 3 [30]. Nevertheless, there is still potential for a higher accuracy, as neither of the two mixing rules are applicable for all blends.

**Table 8:** MAE (left value, in g/10 min), MRE (middle value, in %) and Pearson $R^{2}$ (right value) of recyclate mixtures r16-vH4 and r27-vH4 for model 3 and model 4.

Blend	Model 3			Model 4		
r16-vH4	0.47	5.45	0.995	**0.16**	**1.82**	**0.999**
r27-vH4	0.71	5.62	0.994	**0.23**	**2.02**	**0.998**

## 4. Modeling

### 4.1. Symbolic Regression Analysis

To improve the accuracy in the prediction of the MFR of binary blends, we developed a new rule of mixture based on our experimental data, using symbolic regression based on genetic programming. The dataset used for model construction included the experimental MFR values of our sample blends defined by (i) the MFRs of the individual blending partners and (ii) their corresponding weight fractions in the underlying mixture (see Table 3). Note that in the production of the mixtures, the flowability of the first component $MFR_{1}\;$ was changed from 8.5 to 28.5 g/10 min, whereas the second component $MFR_{2}\;$ was fixed to 4 g/10 min. The latter can hence be regarded as a constant.

In total, 132 data points were considered for model construction, which were randomly subdivided into a training and test set of 64 design points. In contrast to classical regression techniques such as linear or polynomial regression, symbolic regression is a type of analysis that constructs models in the form of mathematical expressions without pre-defining a specific model structure. As the search space for candidate models can become very large, symbolic regression problems are commonly solved by heuristic methods such as genetic programming [43,44]. The latter is an iterative population-based evolutionary algorithm, which creates a population of random mathematical models and refines them iteratively. The usefulness of the modeling approach was demonstrated in various polymer-processing problems [45,46,47,48,49,50].

For regression analysis, we applied the offspring selection genetic algorithm (OSGA) implemented in the open-source software HeuristicLab (Hagenberg, Austria) [51,52] to develop a new rule of mixture in the form of:

(4) $$MFR_{mix}=f\left(MFR_{1},MFR_{2},x_{1},x_{2}\right).$$

This method optimizes the quality of models describing the data only without taking into account model complexity. To specify the search space, we restricted (a) the model size to a maximum tree length of 40 and (b) the function set, which defines the functions applied to generate candidate models, to (i) constant, (ii) variable, (iii) addition, (iv) multiplication and division, and (v) natural logarithm. Model optimization was driven by a constant optimization evaluator, which calculated Pearson $R^{2}$ of a symbolic regression solution (Equation (3)) and optimizes the constant used. Using the training and test data, we performed 10 runs to generate a set of symbolic regression solutions. To evaluate the most accurate approximation, we carried out an error analysis for both subsets.

### 4.2. Symbolic Regression Results

Our modeling approach provided an analytical regression (MTF model) for the MFR of the mixture, where $A_{1}$ to $A_{5}$ are the subfunctions, which contain 13 coefficients. The subfunctions and their coefficients are given in Appendix A. The model includes only basic arithmetical operations and the natural logarithm. The applicability range of the model is restricted to the following MVR ranges of the mixing partners: (i) $8.5\leq MFR_{1}\leq 28.5$ g/10 min and (ii) $MFR_{2}=4$ g/10 min.

(5) $$ln\left(MFR_{mix}\right)=c_{00}+\frac{A_{1}+x_{1}\;ln\left(MFR_{1}\right)\left[c_{03}+A_{2}+A_{3}\right]+A_{4}}{A_{5}}$$

**Figure 10:**
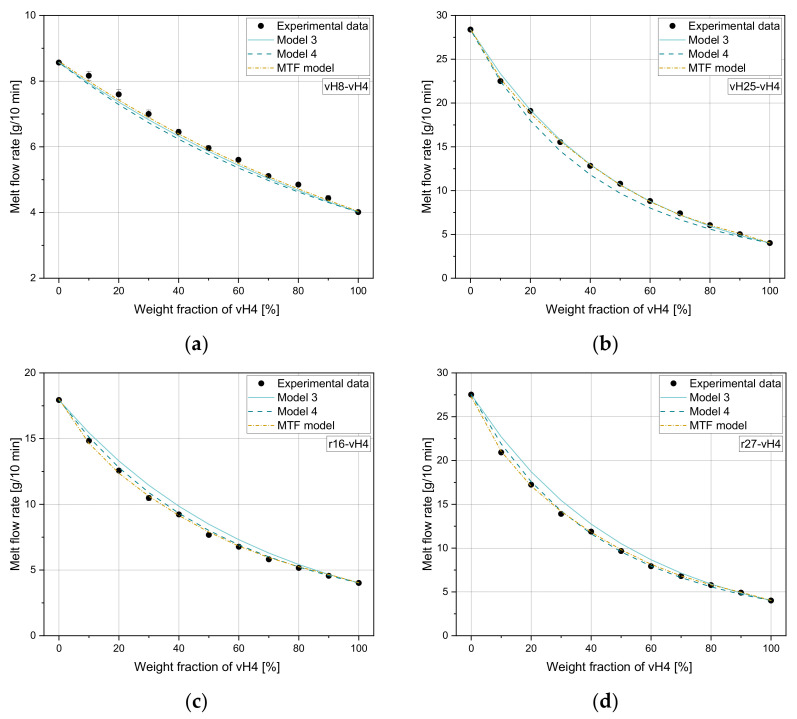
Application of model 3, model 4 and MTF mixing rule on (**a**) vH8-vH4, (**b**) vH25-vH4, (**c**) r16-vH4, and (**d**) r27-vH4.

To demonstrate the performance of the regression model, Figure 10a–d show a comparison of experimental and calculated MVR values for selected mixtures including homopolymers and recyclates. In addition, the results according to the models 3 and 4 are included. For all combinations, the MTF model outperforms the accuracy of the reference models, as additionally demonstrated in Table 9, which indicates the MAE, (ii) the MRE, and (iii) $R^{2}$ for the selected points.

**Figure 11:**
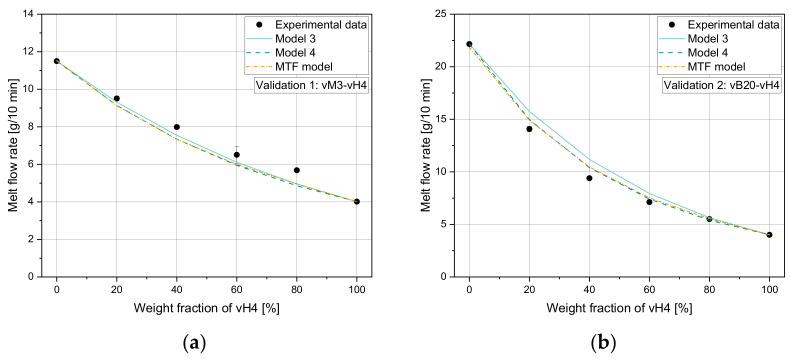
Application of model 3, model 4, and MTF mixing rule on (**a**) vM3-vH4 and (**b**) vB20-vH4.

To validate the new mixing model against unseen experimental data, we used the experimental results obtained for our artificial blends defined in Table 2 (Set 4). A comparison of experimental and calculated results for the mixtures vM3-vH4 and vB20-vH4 is illustrated in Figure 11. The corresponding error measures are listed in Table 10. Note that the design points of the validation set were not used for model development and hence enabled an unbiased estimation of model quality.

**Table 9:** MAE (left value, in g/10 min), MRE (middle value, in %), and Pearson $R^{2}$ (right value) of mixtures vH8-vH4, vH25-vH4, r16-vH4, and r27-vH4 for model 3, model 4, and MTF mixing rule.

Blend	Model 3			Model 4			MTF		
vH8-vH4	0.13	2.04	0.997	0.18	2.94	0.996	0.08	1.35	0.998
vH25-vH4	0.17	1.43	0.999	0.59	5.70	0.997	0.13	1.04	1.000
r16-vH4	0.47	5.45	0.995	0.16	1.82	0.999	0.12	1.47	0.999
r27-vH4	0.71	5.62	0.994	0.23	2.02	0.998	0.13	1.07	1.000

**Table 10:** MAE (left value, in g/10 min), MRE (middle value, in %), and $R^{2}$ (right value) of mixtures vM3-vH4 and vB20-vH4 for the mixing rules model 3, model 4, and MTF.

Blend	Model 3			Model 4			MTF		
vM3-vH4	0.29	4.44	0.991	0.40	5.84	0.986	0.38	5.52	0.987
vB20-vH4	0.74	7.51	0.985	0.39	3.89	0.995	0.44	4.15	0.994

**Figure 12:**
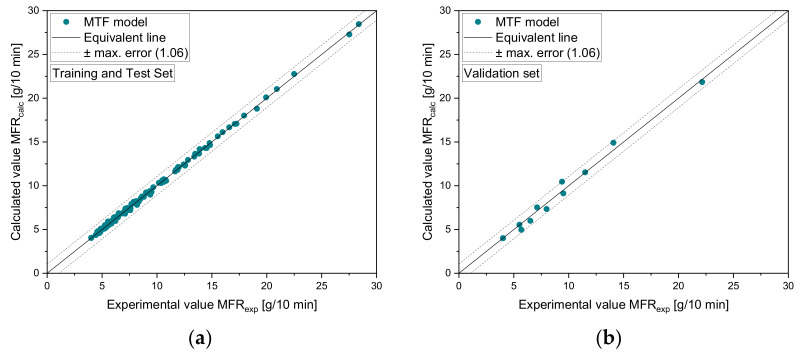
Scatter plots of MTF model: (**a**) training and test set and (**b**) validation set. The dashed lines indicate an absolute error of 1.06.

To demonstrate the prediction accuracy of the new MTF model for the (i) training, (ii) test, and (iii) validation sets, Figure 12 shows a normalized representation of all design points in the form of scatter plots that compare experimental and calculated results for all subsets. The MAE of the MTF model for the subsets 1 to 3 is 0.36, 0.42, and 0.27 g/10 min, respectively. For subset 4 (validation set), a mean error of 1.06 g/10 min was calculated.

## 5. Discussion

Flowability adjustment by the use of mixing rules is a widely used method when it comes to viscosity adjustment of crude oil [32,34]. The application of mixing rules for adjusting the MFR of re-granules via compounding with low MFR grades is a practicable method to achieve more uniform recyclate compounds and therefore higher product quality. While conventional mixing rules such as the frequently used Arrhenius mixing rule (model 3) achieve good agreement for blends of virgin polymers, for recyclates Cragoe’s mixing rule (model 4) delivers more accurate results with a maximum relative error of 5.70%, which was shown in the Results. However, both mentioned rules lack an accurate applicability for all polymer types used in this research work. A rule which reaches low error for blends of one virgin partner with a pre-defined MFR with (i) virgin, (ii) artificial recyclate, and (iii) recyclate materials was not found within the selected mixing rules. Therefore, the MTF mixing rule was established. From the overall perspective, with this predictive model a higher accuracy for all tested blends was achieved in the considered data ranges. The maximum relative errors were 3.40% and 5.52% for the training and test set and the validation set, respectively. In contrast, maximum relative errors of 7.51% and 5.84% for the mixing rules of Arrhenius and Cragoe, respectively, were determined in the validation set. Table 11 shows that the MTF mixing rule outperforms both existing mixing rules according to Arrhenius and Cragoe with an overall lower MAE and MRE considering sample sets 1 to 3. Compared to crude oils, recyclates are a complex blend from various waste streams with several contaminations, and it was expected that an existing mixing rule cannot describe all of the data. Hence, a tailored mixing rule was developed.

In terms of continuous polymer processing, well-adjusted MFR values in polyolefin recyclate grades are essential to enable constant processing and good-quality products. When it comes to products of virgin polymer grades, these grades are developed for certain processing methods and for specific applications. Hence, their property profile is well defined and constant, independent of the material batch. However, even high-quality post-consumer recyclate grades are always a blend of plastics waste out of products of various applications and produced by different processing methods. The mixture of the whole plastic waste of one polymer type leads to averaged rheological and mechanical properties. To ensure the production of recyclates that are within a certain specification, it is of utmost importance that at least the MFR is well-adjusted for its respective field of application allowing for constant processing without errors and defects. For this purpose, mixing rules can be used to enable constant melt flow rates even when the recycling streams have fluctuating melt flow properties. Furthermore, mixing rules can be used, if the best fitting recyclate is not available on the market and therefore the MFR values of the available recyclates can be adjusted by mixing with a virgin polymer grades.

## 6. Conclusions and Outlook

This paper deals with the evaluation of the applicability of mixing rules on polypropylene (PP) mixtures using three datasets of (i) virgin mixtures, (ii) artificial recyclates, and (iii) commercial recyclates. Six selected mixing rules were tested by comparing the calculated values with experimentally determined melt flow rate (MFR) results. The mixing rules of Arrhenius and Cragoe showed the smallest deviations from the measured results, with Cragoe’s mixing rule being more suitable for recyclates. In the next step, a new mixing rule, called the MTF mixing rule, was developed using symbolic regression. Although this mixing rule is more complex than the existing ones, a higher overall agreement with the experimental values was observed for all measured values. Finally, the MTF model was validated by two additional series of measurements in previously uncovered MFR ranges, and the accuracy of this new developed mixing rule was confirmed.

In further research, the applicability of selected mixing rules and the MTF model will be tested on additional PP mixtures to make it generally applicable. The variation in the second component, which was chosen to be constant in this publication, and the determination of the influence of different PP types such as random and block copolymers, additionally to homopolymers, are of particular interest. Furthermore, other polymers apart from PP will be investigated.

## Figures and Tables

**Table 1:** Overview of materials including designation and MFR value (in g/10 min; 230 °C/2.16 kg).

Designation	Material	MFR	Designation	Material	MFR
vH4	HC205TF	4	vB8	BD310MO	8
vH8	HD120MO	8	vB20	BF970MO	20
vH12	HE125MO	12	r16	Moplen QCP300P	16
vH20	HF955MO	20	r27	Purpolen PP	25
vH25	HG385MO	25			

**Table 2:** Description and blending partner composition of sample blends (in weight percentage).

Set	Blend	MFR_1_									MFR_2_
		vH8	vH12	vH20	vH25	vB8	vB20	r16	r27		vH4
1	vH8-vH4	100	-	-	-	-	-	-	-	+	100
	vH12-vH4	-	100	-	-	-	-	-	-		
	vH20-vH4	-	-	100	-	-	-	-	-		
	vH25-vH4	-	-	-	100	-	-	-	-		
2	vM1-vH4	25	25	25	25	-	-	-	-		
	vM2-vH4	20	20	20	20	20	-	-	-		
3	r16-vH4	-	-	-	-	-	-	100	-		
	r27-vH4	-	-	-	-	-	-	-	100		
4	vM3-vH4	50	50	-	-	-	-	-	-		
	vB20-vH4	-	-	-	-	-	100	-	-		

**Table 3:** Weight fractions $x_{1}$ and $x_{2}$ for each material mixture.

Mixture	1	2	3	4	5	6	7	8	9	10	11
$x_{1}$	100	90	80	70	60	50	40	30	20	10	0
$x_{2}$	0	10	20	30	40	50	60	70	80	90	100

**Table 4:** Overview of selected mixing rules for polymer blends [30,32,34,35,36,37].

No.	Model	Equation	Source
1	Linear	$MFR_{mix}=x_{1}\;MFR_{1}+x_{2}\;MFR_{2}$	[38]
2	K & M	$MFR_{mix}{}^{\frac{1}{n}}=x_{1}\;MFR_{1}{}^{\frac{1}{n}}+x_{2}\;MFR_{2}{}^{\frac{1}{n}}$	[39]
3	Arrhenius	$\ln\left(MFR_{mix}\right)=x_{1}\ln\left(MFR_{1}\right)+x_{2}\ln\left(MFR_{2}\right)$	[35]
4	Cragoe	$\frac{1}{\ln\left(L\;MFR_{mix}\right)}=\frac{x_{1}}{\ln\left(L\;MFR_{1}\right)}+\frac{x_{2}}{\ln\left(L\;MFR_{2}\right)}$	[37]
5	Walther	$\ln\ln\left(MFR_{mix}+C\right)=x_{1}\ln\ln\left(MFR_{1}+C\right)+x_{2}\ln\ln\left(MFR_{2}+C\right)$	[40]
6	Bingham	$MFR_{mix}{}^{-1}=x_{1}\;MFR_{1}{}^{-1}+x_{2}\;MFR_{2}{}^{-1}$	[41]

**Table 5:** MAE (left value, in g/10 min) and MRE (right value, in %) of virgin mixtures for all tested mixing rules.

Blend	Model 1		Model 2		Model 3		Model 4		Model 5		Model 6	
vH8-vH4	0.14	2.37	**0.06**	**0.94**	0.13	2.04	0.18	2.94	0.27	4.41	0.38	6.09
vH12-vH4	1.16	15.46	0.52	6.52	0.20	2.28	**0.11**	**1.28**	0.32	4.24	0.71	9.05
vH20-vH4	2.09	24.31	0.87	9.53	0.25	2.62	**0.12**	**1.35**	0.58	6.26	1.42	14.25
vH25-vH4	3.43	32.17	1.20	9.93	**0.17**	**1.43**	0.59	5.70	1.34	11.84	2.86	22.73

**Table 6:** Pearson $R^{2}$ of virgin mixtures for all tested mixing rules.

Blend	Model 1	Model 2	Model 3	Model 4	Model 5	Model 6
vH8-vH4	0.992	**0.998**	0.997	0.996	0.990	0.979
vH12-vH4	0.959	0.990	0.996	**0.997**	0.993	0.973
vH20-vH4	0.941	0.989	0.999	**0.999**	0.993	0.955
vH25-vH4	0.931	0.989	**0.999**	0.997	0.985	0.918

**Table 7:** MAE (left value, in g/10 min), MRE (middle value, in %), and Pearson $R^{2}$ (right value) of artificial recyclate mixtures for model 3 and model 4.

Blend	Model 3			Model 4		
vM1-vH4	0.19	2.09	0.998	**0.12**	**1.61**	**1.000**
vM2-vH4	**0.18**	**2.01**	**0.998**	0.27	3.63	0.996

**Table 11:** Errors for data points: MAE (left, in g/10 min), MRE (middle, in %), and Pearson $R^{2}$ (right) for Arrhenius, Cragoe, and MTF mixing rule.

Rule of Mixture	MAE	MRE	R^2^
Arrhenius (model 3)	0.29	3.29	0.995
Cragoe (model 4)	0.22	2.67	0.997
MTF	0.16	2.02	0.997

## Data Availability

The data presented in this study are available on request from the corresponding author.

## Appendix A

### Appendix A.1. Subfunctions of Equation (4) with $MFI_{2}=4g/10\;\min$

$A_{1}=\frac{1}{c_{01}+c_{02}\;ln\left(MFI_{1}\right)}$	(A1)
$A_{2}=\frac{c_{04}}{ln\left(MFI_{2}\right)+c_{05}\;x_{1}ln\left(MFI_{1}\right)}$	(A2)
$A_{3}=\frac{c_{06}\;\;ln{\left(MFI_{1}\right)}^{2}\;\frac{x_{2}}{ln\left(MFI_{2}\right)}}{c_{07}+c_{08}\;ln\left(MFI_{1}\right)+c_{09}\;\;ln{\left(MFI_{1}\right)}^{2}-ln{\left(MFI_{1}\right)}^{3}}$	(A3)
$A_{4}=c_{10}\;x_{2}\;ln\left(MFI_{2}\right)$	(A4)
$A_{5}=c_{11}+c_{12}\;\frac{x_{2}}{ln\left(MFI_{2}\right)}$	(A5)

### Appendix A.2. Model Coefficients

**Table A1:** Model coefficients.

$c_{00}$	0.0243327	$c_{07}$	26.6035
$c_{01}$	6292.96	$c_{08}$	−26.8233
$c_{02}$	−2934.41	$c_{09}$	8.98627
$c_{03}$	−4.80228	$c_{10}$	−5.77251
$c_{04}$	17.9609	$c_{11}$	−4.92978
$c_{05}$	−88.3534	$c_{12}$	−1.313
$c_{06}$	0.0000276798		
